# Prognosis of Biomarker of Alzheimer's Disease in the Function of the Retina and Secondary Molecular Structure Variation of the Retina and Brain

**DOI:** 10.1155/2023/9775921

**Published:** 2023-03-29

**Authors:** Heba Ahmed Gaber, Eman Mohamed Aly, Eman Saad Mohamed, Marwa Elfouly, Mona Salah Talaat, El-Sayed Mahmoud El-Sayed

**Affiliations:** ^1^Biophysics and Laser Science Unit, Visual Science Department, Research Institute of Ophthalmology, Giza, Egypt; ^2^Ophthalmology Department, Research Institute of Ophthalmology, Giza, Egypt; ^3^Biophysics Department, Science College, Ain Shams University, Cairo, Egypt

## Abstract

Alzheimer's disease (AD) is one of the most serious neurodegenerative diseases in the globe. As a result, there is an acute need to discover indications that allow for early disease detection. There is growing scientific data showing the similarities between the eye and other central nervous system components, suggesting that information obtained in ophthalmic research might be valuable in the study and diagnosis of AD. Fifty male albino Wistar rats were separated into five groups: the first group served as control, and the other four groups of animals were administrated aluminium chloride (AlCl_3_) in a dose of 100 mg/kg body weight (b.w.) for 2, 4, 6, and 8 weeks, respectively. Insights into the function of the retina by electroretinogram (ERG) and the changes thought to have occurred in the molecular structure of the retina and brain using Fourier transform infrared spectroscopy (FTIR) as a result of AD progression induced by AlCl_3_ in rats were done. Moreover, the measurement of acetylcholinesterase (AchE) was done. After 6 and 8 weeks of AlCl_3_ injection, there was a substantial reduction (*p* ≤ 0.05) in a- and b-wave amplitudes and a significant rise (*p* ≤ 0.05) in implicit time compared to controls. A significant elevation (*p* ≤ 0.05) of AchE content was observed after 4, 6, and 8 weeks. FTIR revealed a significant increase (*p* ≤ 0.05) of *β*-turn and *β*-sheet content associated with significant decrease (*p* ≤ 0.05) of *α*-helix content for all groups administrated with AlCl_3_. Our findings suggest that retinal biomarkers such as ERG of the retina may be used as a screening tool for detection of AD. Secondary structural changes in the proteins of the retina and the brain were similar in AD rats' model and precede retinal dysfunction.

## 1. Introduction

Alzheimer's disease (AD) is the most frequent neurodegenerative confusion in the elderly. AD involves 11% of the population over the age of 65 and almost half of people aged 85 years and older. There is, however, no definite early diagnostic indicator, no effective disease-modifying therapy, and no effective prophylaxis for AD [1].

The precise root cause of AD is yet unknown. Numerous studies revealed associations between certain risk factors and the development of AD, including depression, ageing, head trauma, oxidative stress, neuroinflammation, and persistent exposure to environmental metal toxicants [2, 3].

There is also mounting evidence that neurological disorders are associated with metal toxicity, including lead, cadmium, and aluminium, with aluminium being the most effective neurotoxin [4]. The brain is a prime target for aluminium poisoning, and due to its high affinity for receptors, it might easily traverse the blood-brain barrier (BBB) and finally accumulate in the brain [5].

The growth of intracellular neurofibrillary knots and the buildup of amyloid beta peptide (A*β*) in neuronal cells are considered the main histopathological features of AD. Additionally, acetylcholine (Ach) breakdown, which is a key component of healthy memory and cognition, is elevated by A*β* overexpression [6].

Since both the retina and the brain are components of the central nervous system, they share an embryological origin. Age-associated retinal neurodegenerative illnesses and brain neurodegenerative diseases, including AD, are currently recognised to be connected [7]. Additionally, protein deposits in the retina have been found in AD animal models as well as in vivo and postmortem eyes from human AD patients, with the retina having significant diagnostic implications for AD [8].

Visual symptoms have also been recorded in the early stages of AD, even before the diagnosis is definitively made, along with cognitive problems. Patients with AD show abnormalities in their visual acuity [9], contrast sensitivity [10], colour vision [11], and motion perception [12], according to visual exams. Indeed, growing data suggests that retinal modifications such retinal ganglion cell (RGC) degeneration and loss [13], decreased thickness of the retinal nerve fibre layer (RNFL) [14, 15], and decreased retinal blood flow [16] may contribute to visual dysfunctions in AD patients.

The development of treatments for the later stages of Alzheimer's has not been effective, and it seems likely that future pharmaceutical treatments will be more effective in the early stages of the disease. Furthermore, because diagnostic tests for AD are invasive or difficult to access from outside research institutes, there has been a movement towards the development of more accessible biomarkers. This opens up the possibility of using the retina as a biomarker for cortical diseases such as dementia. In this study, we provided insight into the function of the retina by electroretinogram (ERG) and the changes thought to have occurred in the molecular structure of the retina and brain using Fourier transform infrared spectroscopy (FTIR) as a result of AD progression induced by AlCl_3_ in rats for the first time. Moreover, measurement of acetylcholinesterase (AchE) in the brain was done.

## 2. Materials and Methods

### 2.1. Chemicals

All chemicals were bought from Sigma-Aldrich Company (St. Louis, MO, USA).

### 2.2. Animals

Fifty male albino Wistar rats (200-250 g) were used in this study. Animals were taken from the animal house of the Research Institute of Ophthalmology (RIO) where all experiment techniques were carried out. The rules of the Association for Research in Vision and Ophthalmology (ARVO), the recommendations of the local research committee of RIO, and ARRIVE guidelines were followed. The approval number from the local ethical committee is FWA 00031860. Rats were kept in a typical setting of a 12-hour lighting cycle and a temperature of 25°C; they had unrestricted access to water and chewing a food. Following a week of acclimation, rats were separated into five groups of 10 animals each. The first group served as control, and the other four groups of animals were administrated AlCl_3_ in a dose of 100 mg/kg body weight (b.w.) [17] for 2, 4, 6, and 8 weeks, respectively. The Morris water maze test was done and revealed a significant memory and learning deficits after 6 weeks of AlCl_3_ administration, indicating that AD was established [18].

### 2.3. ERG Recording

For three hours prior to the electrophysiological recording, the animals were dark-adapted. Animals were put on an operational table cushion with their body temperatures kept at 37°C. xylazine (21 mg/kg body weight) was used as a muscle relaxant, and ketamine hydrochloride (45 mg/kg) was also administered intramuscularly to the rats [19]. Eye drops that provide temporary anaesthesia were also used. Topical 1% Mydriacyl was used to dilate the pupil of the eye being monitored. In this work, a white flash with a predetermined intensity of 4 lux and duration of 0.2 seconds was employed. The ERG was captured using the PASCO, Roseville, CA, electrodes which connect directly to the computer via the PASPORT and sensor PS-2111. The active electrode was positioned at the corneal edge, and the reference electrode was positioned on the lower eyelid skin. The last one electrode that is earthed was placed on the ear. Data Studio 1.9.8 software was used to analyze the signals that occurred.

### 2.4. FTIR Measurements

After ERG measurements, the animals were killed, and performing an eye enucleation, the corneal section was used to open the eye, exposing the retina and allowing the anterior segment components to be removed. After one hour of freeze-drying, the retinae were combined with the potassium bromide (KBr) discs that will be utilized for the FTIR analysis with KBr powder (2 mg retina: 98 mg KBr).

At certain periods, rats were euthanized by intraperitoneal injection of 800 mg/kg sodium pentobarbital [20]; the brain hippocampus tissues were subsampled from rats, homogenated, and placed immediately in liquid nitrogen, and then, KBr discs were performed to do FTIR analysis.

To get better the signal-to-noise ratio, the FTIR spectra were acquired using a Shimadzu FTIR spectrometer that has been continuously supplied dry nitrogen gas to eliminate the influence of water vapor and ambient carbon dioxide (CO_2_). Each sample received one hundred interferograms. Before applying Savitzky-Golay method's Fourier transform, these interferograms were subsequently coadded, baseline adjusted, and flattened. The average group spectrum was created by averaging the spectra from each group using the software OriginPro 9. The method for curve enhancement—a combination of nonlinear curve fitting and Fourier deconvolution—was used to this final average group spectrum to resolve the shape of the amide I band (1750-1600 cm^−1^) towards its constituent peaks. The second derivative of the group spectrum confirmed the number of underlying peaks that occurred [21].

### 2.5. AchE Content Measurement

The levels of AchE in the brain tissues of control and treated animals were determined according to the method of [22]. AchE is a highly viable target for the AD, and cholinergic variation is a consistent and early finding in AD [23]. Hippocampus from treated animals was suspended in 0.25 M sucrose buffer and held for 30 min. The samples were next centrifuged at 10,000 revolutions per minute (rpm), and the supernatant was used to assay the AchE content by a spectrophotometric technique. Absorbance was measured at 412 nm, and the results are presented as nanogram per gram tissue.

### 2.6. Statistical Analysis

Data were expressed as mean ± standard deviation (SD). Analysis of variance (ANOVA) was used to compare between all studied groups. The results were considered significant at *p* < 0.05 using a commercially available software package (SPSS-11, for Windows). For more statistical adjustment to avoid the possibility of type I error, the Bonferroni correction was applied where the significant level of the ANOVA test (*p* < 0.05) was lower than 0.00625. OriginPro 9 software (OriginLab Corporation, Northampton, MA, USA) was used to do multivariate analysis and principal component analysis (PCA).

## 3. Results

### 3.1. ERG

**Figure 1:**
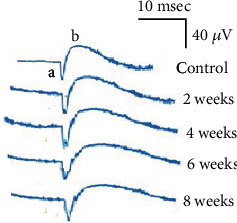
The ERG responses of the dark-adapted eyes for the control group and rats administrated aluminium chloride for 2, 4, 6, and 8 weeks.

The responses of ERG to the dark-adapted eyes of the control group and rats administrated AlCl_3_ for 2, 4, 6, and 8 weeks are illustrated in Figure 1.  Table 1 shows the characterization characteristics of a- and b-waves (amplitude and implicit time) for all groups. Amplitudes for the a-wave were determined from the base to the lowest level of the negative peak and for the b-wave from the latter to the positive peak. For control, the implicit time of a-wave is 13.2 ± 0.9 m sec and the amplitude is 26.1 ± 0.9 *μ*V, while for the b-wave, they are 34.4 ± 0.7 m sec and 54.8 ± 1.8 *μ*V, respectively. It was noticed that there were no changes in a- and b-wave amplitude or implicit time compared to control after 2 and 4 weeks of AlCl_3_ administration. A substantial reduction (*p* < 0.05) in a- and b-wave amplitudes was observed after 6 and 8 weeks of AlCl_3_ administration. In contrast, the implicit time indicated a significant increase (*p* < 0.05) for the same groups compared to the control.

### 3.2. AchE

**Figure 2:**
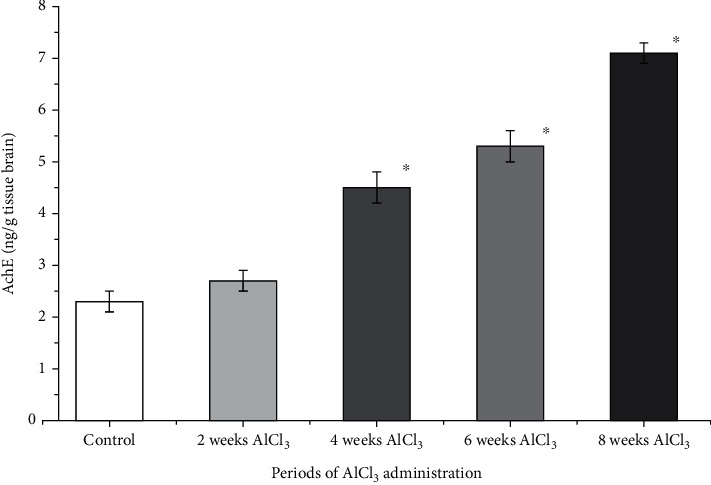
Histogram for the brain content of AchE in rats given 100 mg/kg b.w. of AlCl_3_ after 2, 4, 6, and 8 weeks compared to control.

Figure 2 indicates the histogram for the brain content of AchE in control rats, and rats were given 100 mg/kg b.w. of AlCl_3_. AchE levels in normal brain hippocampus tissue were 2.3 ± 0.1 ng/g tissue brain. A significant elevation (*p* < 0.05) of AchE content was observed after 4, 6, and 8 weeks of AlCl_3_ administration compared to control.

### 3.3. FTIR of the Retina

**Figure 3:**
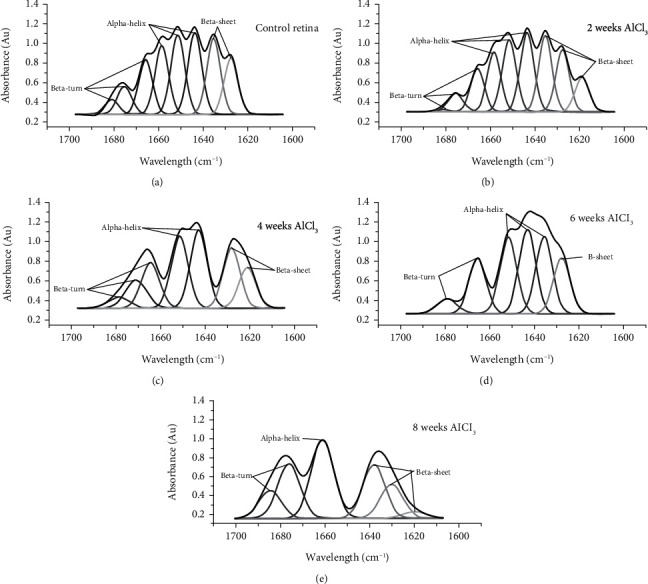
FTIR spectra (1700-1600 cm^1^) of amide I band of the retinal tissue after deconvolution for the control rat group (a) and all groups administrated aluminium chloride after 2 weeks (b), 4 weeks (c), 6 weeks (d), and 8 weeks (e).

Figures 3(a)–3(e) illustrate the FTIR spectra in the 1700-1600 cm^1^ range that were related to the amide I band of the retinal tissue after deconvolution to resolve the contour of amide I in different structural components of protein for the control rat group (Figure 3(a)) and all groups administrated AlCl_3_ after 2 weeks (Figure 3(b)), 4 weeks (Figure 3(c)), 6 weeks (Figure 3(d)), and 8 weeks (Figure 3(e)). The control spectra (Figure 3(a)) show eight structural components of proteins detectable at 1681 ± 1 cm^−1^, 1675 ± 2 cm^−1^, and 1666 ± 1 cm^−1^ (*β*-turn); 1659 ± 1 cm^−1^, 1651 ± 1 cm^−1^, and 1643 ± 1 cm^−1^ (*α*-helix); and 1635 ± 1 cm^−1^ and 1626 ± 1 cm^−1^ (*β*-sheet). Due to the administration of AlCl_3_ over 2, 4, 6, and 8 weeks, the number of bands changes to nine, seven, six, and six bands, respectively, as shown in Figures 3(a)–3(e).

Table 2 illustrates the percentages of area under the peaks of all retinal structural protein components (*β*-turn, *α*-helix, and *β*-sheet) for all groups that were administrated AlCl_3_ compared to a control. A significant increase (*p* < 0.05) of *β*-turn and *β*-sheet contents was associated with a significant decrease (*p* < 0.05) of *α*-helix content for all groups administrated AlCl_3_, except *β*-turn content for group 2 weeks.

### 3.4. FTIR of the Brain

**Figure 4:**
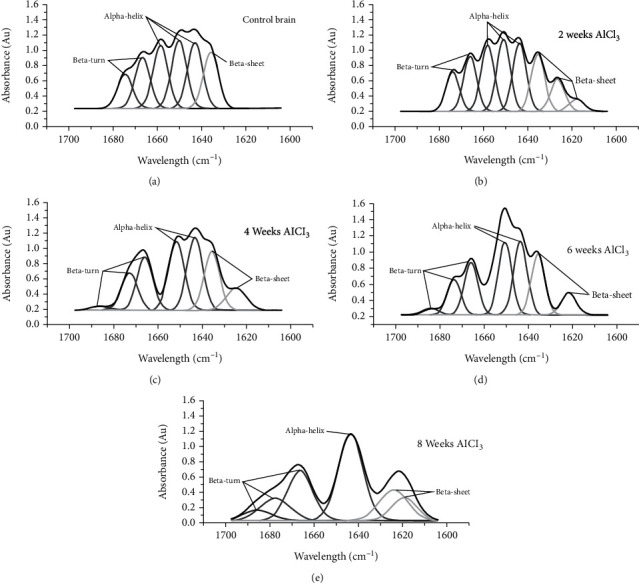
The deconvolution spectra of FTIR due to amide I (1700-1600 cm^−1^) of the brain's hippocampus tissue from control rats (a) and groups that received aluminium chloride after 2 weeks (b), 4 weeks (c), 6 weeks (d), and 8 weeks (e).

Figures 4(a)–4(e) show the deconvolution spectra of FTIR due to amide I (1700-1600 cm^−1^) of the brain's hippocampus tissue from control rats (Figure 4(a)) and groups that received AlCl_3_ after 2 weeks (Figure 4(b)), 4 weeks (Figure 4(c)), 6 weeks (Figure 4(d)), and 8 weeks (Figure 4(e)). The deconvolution spectra of the control brain showed six bands caused by various protein structural components. These bands have the following assignments: *β*-turn (1675 ± 2 and 1667 ± 2 cm^−1^), *α*-helix (1659 ± 2, 1650 ± 2, and 1643 ± 1 cm^−1^), and *β*-sheet (1635 ± 2 cm^−1^).

The percentages of area under the peaks for the main protein structures of brain tissue in all groups administrated AlCl_3_ compared to control brain rats are calculated and listed in Table 3. All identified protein structure content showed a significant decrease (*p* < 0.05) for *α*-helix, and a significant increase (*p* ≤ 0.05) for all other protein structures except *β*-turn after 2 weeks of AlCl_3_ administration did not show a significant change.

### 3.5. Principal Component Analysis

**Figure 5:**
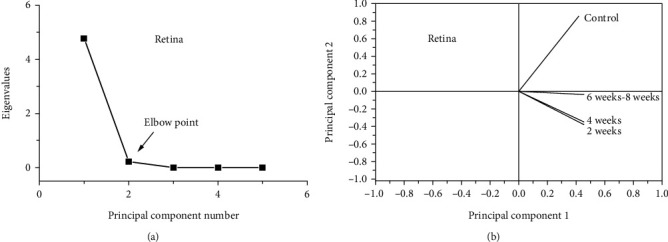
(a) Eigenvalues in relation to the principal components of FTIR data and (b) the loading plot for retinal amide I data.

Figures 5(a) and 5(b) depict the eigenvalues in relation to the principal components (Figure 5(a)) and the loading plot for retinal amide I raw data (Figure 5(b)). The covered data was 99.93% due to 95.60% for the first principal component and 4.30% for the second principal component as shown in Figure 5(a). Figure 5(b) reveals a complete contrast between the control group and all animal groups given AlCl_3_ (2, 4, 6, and 8 weeks), because the control group has loading on PC2 and all AlCl_3_ groups have loading on PC1.

**Figure 6:**
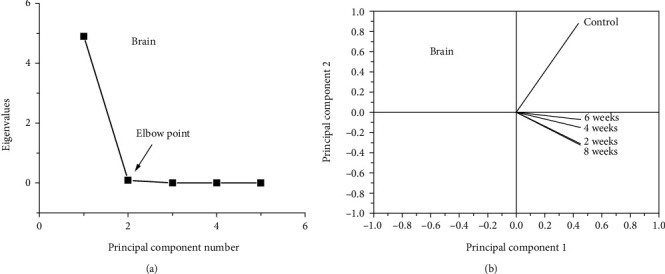
(a) Eigenvalues in relation to the principal components of FTIR data and (b) the loading plot of PCA for brain amide I FTIR data.

Figures 6(a) and 6(b) depict the eigenvalues in relation to the principal components (Figure 6(a)) and the loading plot for raw data from the brain FTIR spectra (Figure 6(b)). The covered data was 99.84% due to 98.13% for the first principal component and 1.71% for the second principal component (Figure 6(a)). Figure 6(b) reveals the same phenomenon observed in retinal data analysis, i.e., complete contrasts between the control group and all animal groups administered AlCl_3_ (2, 4, 6, and 8 weeks); the control has a load on PC2, and all AlCl_3_ groups have a load on PC1.

## 4. Discussion

Aluminium chloride model is increasing due to its relationship with the neurotoxicity of AD. This model exposed that rats are harmonious with the main pathological features of AD such as oxidative stress, inflammation, neuron death, and cholinergic degradation. Thus, the AD model used in this study meets the experimental AD requirements. Several studies have used the dose of 100 mg/kg b.w. of AlCl_3_ for 6 weeks that was considered sufficient to accelerate the process of neurodegeneration in animal models [17, 24, 25].

Results of the current study indicated that administration of AlCl_3_ led to a significant increase (*p* < 0.05) of AchE content in the brain starting from 4 weeks and that is ascribed to the direct effect of AlCl_3_. According to Zatta et al. [26], AlCl_3_ may influence AchE activity by reacting with its peripheral locations and changing the secondary structure. The cholinergic hypothesis is the earliest significant theory about the pathogenesis of AD [27]. Acetylcholine (ACh) is a crucial neurotransmitter implicated in memory and learning processes, as well as changes in cholinergic activity, which is the primary event in the neurochemistry changes associated with AD. AchE is also considered a cholinergic activity marker enzyme that destroys and ends ACh's physiological effect. Each molecule of AchE degrades approx 25,000 ACh molecules per second in both neural and nonneural tissues. Acetylcholinesterase binds directly to presenilin-1 (PS-1), an essential enzyme in the A*β* synthesis pathway, and increases its expression, raising the amount of A*β* and accelerating cognitive impairment [28]. Moreover, improper central cholinergic alterations may result in abnormal tau protein phosphorylation, neurotransmitter and neurohormone system instability, nerve cell inflammation, cell death, and other pathogenic occurrences. Clinical findings demonstrate that the brains of patients with AD have significant neurodegeneration, a loss in cholinergic neurons, and a severe ACh deficit, demonstrating the impairment to the cholinergic system in individuals with AD following acute injury [29].

The ERG is a noninvasive eye exam that measures electrical activity in the retina. In this study, we examined the development of retinal function in an AlCl_3_ model of AD in rats compared to a control using ERG. The changes in decrease in a- and b-wave amplitude or increase in implicit time were significant only after 6 and 8 weeks of aluminium chloride administration. This demonstrates that retinal dysfunction occurs after changes in the brain AchE in the AD rat model. The pattern of retinal neurodegeneration is similar to that of the brain. As the disease progressed, functional failure of the outer retina occurred [30]. Early-stage AD is characterized by a loss in synaptic proteins, which might explain the deterioration in retinal function. In neurodegenerative diseases such as AD, aberrant calpain activation promotes accumulation of A*β* buildup and tau hyperphosphorylation in neurons and is related with synaptic dysfunction [31]. In AD, synaptic dysfunction is intimately linked to oxidative stress [32]. Oxidative stress-induced neurotoxicity is a major pathologic outcome of the primary neurodegenerative process of AD. According to a study by Liu et al. [33], 3xTg AD mice exhibited a substantial reduction in scotopic b-wave as evaluated by flash ERG compared to WT mice.

FTIR is an advanced technique capable of characterizing oxidative stress markers associated with protein denaturation. One of the most essential and complicated organs is the human brain. Each unique diverse anatomical area is extremely specialized. Electrical impulses are generated by nerve cells and move across the body via excitable semipermeable membranes that modify the permeability of tiny molecules. Any molecular alteration in nerve tissue might cause malfunction in any region of the body.

For the first time, an effort was made in this work to discover a spectroscopic marker of AD utilizing FTIR spectra of the brain and retina in the region related to amide I (1700-1600 cm^−1^). The secondary protein structure is determined using amide I. The range between 1640 cm^−1^ and 1660 cm^−1^ accounts for the intramolecular hydrogen bonding in the C_O⋯H\\N group that creates the *α*-helix shape, whereas the C_O⋯H\\N group's intermolecular hydrogen bonds create *β*-sheet or *β*-turn with IR ranges of 1640 and 1620 cm^−1^ and 1660–1690 cm^−1^, respectively. Proteins perform the majority of the functions in living cells, and so it must fold to their unique three-dimensional structural. Inefficient function of proteins was due to poor protein folding or insolubility. The results of this study were revealing the early detected secondary structure changes of proteins (*α*-helix and *β*-sheet) in the brain and retina from 2 weeks of AlCl_3_ administration. Interestingly, the changes in the percentage of the content of the main protein secondary structural components (*β*-turn, *α*-helix, and *β*-sheet) are similar to those in the retina and brain.

Administration of AlCl_3_ caused the protein to become aggregated, more folded, and insoluble. This may be drawn from the apparent decrease in *α*-helix content that occurred concurrently with a rise in *β*-sheet content as a result of the development of an intramolecular hydrogen-bonded *β*-sheet structure. According to one theory, the amount of *β*-sheet structure correlates with the amount of insoluble protein [21]. This protein conformational change in the brain was a marker for neurodegeneration that occurred due to AlCl_3_ administration. On the other hand, after 4, 6, and 8 weeks of AlCl_3_ administration, *β*-turn content was increased compared to control. Since *β*-turns are characterized as polypeptide sections where the chain direction changes [34], they have the potential to affect protein stability and actively contribute to the folding process by serving as a nucleation site. As a result, the turn's construction encourages the development of supersecondary structures [35]. Protein aggregation, folding, and denaturation are resulting in loss of function that may clarify ERG variation.

Using PCA, the results added a new value in the assessment of disease progression. Differently administrated AlCl_3_ groups (2, 4, 6, and 8 weeks) have the same load on PC1 for the retina and brain. These results reflect the impact of time on the progress of the disease, where the variation of protein secondary structure of the retina in periods 2-4 weeks differs than that appeared in 6-8 weeks. Unlike the retinal analysis data, PCA of the brain revealed that the brain variation caused by AD is not functional in time.

The retina can exhibit the traditional symptoms of AD because it is a part of the central nervous system. Amyloid precursor proteins may be able to go from retinal ganglion cells to the cortex and vice versa through the optic nerve, which connects the retina and the brain [36]. Additionally, these modifications are responsible for structural changes in the retina as well as many retinal cell atrophy and/or die. Since the retina acts as a “window” to the brain, its eyesight offers a direct and noninvasive technique of detecting the telltale signals of AD without causing significant discomfort to the patient.

In conclusion, retinal biomarkers such as ERG of the retina may be used as a screening tool for the detection of AD in rats. FTIR spectroscopy is a nondestructive and rapid method to characterize oxidative stress markers associated with protein denaturation, accelerating research into AD and proposing new approaches to neurodegenerative diseases. We show for the first time that secondary structural changes in the proteins of the retina and the brain were similar in AlCl_3_ model of AD in rats and precede retinal dysfunction. Further research is required to clarify whether this phenomenon happens in human and whether analyzing structural and functional changes in the retina might assist in the early detection of AD.

## Figures and Tables

**Table 1:** The amplitude (*μ*V) and the implicit time (m sec) for a- and b-wave to all groups administrated aluminium chloride compared to control. The data were expressed as the mean ± SD.

	a-wave		b-wave	
	Amplitude (*μ*V)	Implicit time (m sec)	Amplitude (*μ*V)	Implicit time (m sec)
Control	26.1 ± 0.9	13.2 ± 0.9	54.8 ± 1.8	34.4 ± 0.7
2-week AlCl_3_	25.4 ± 1.9	13.5 ± 0.8	52.97 ± 1.6	33.1 ± 0.3
4-week AlCl_3_	25.2 ± 1.2	14.2 ± 0.3	53 ± 0.6	32.3 ± 0.1
6-week AlCl_3_	20.8 ± 1.1^†^	19.4 ± 0.7^†^	48.6 ± 1.9^†^	45.8 ± 0.2^†^
8-week AlCl_3_	19.6 ± 0.5^†^	23.3 ± 0.6^†^	43.1 ± 0.4^†^	51 ± 0.3^†^

^†^Statistically significant (*n* = 10, *p* < 0.05).

**Table 2:** Curve fitting analysis of amide I bands represents the percentages of area under peak (%) of main protein secondary structural components (*β*-turn, *α*-helix, and *β*-sheet) of rat's retinal tissue in all groups administrated aluminium chloride compared to control.

Retina	*β*-Turn	*α*-Helix	*β*-Sheet
Control	20.4 ± 1	50.1 ± 2	29.5 ± 2
2-week AlCl_3_	19.3 ± 1	44.1 ± 1^†^	36.6 ± 3^†^
4-week AlCl_3_	27.8 ± 1^†^	37.5 ± 2^†^	34.7 ± 1^†^
6-week AlCl_3_	24.3 ± 1^†^	39.8 ± 1^†^	35.9 ± 1^†^
8-week AlCl_3_	32.1 ± 1^†^	30.6 ± 3^†^	37.3 ± 1^†^

^†^Statistically significant (*n* = 10, *p* < 0.05).

**Table 3:** Curve fitting analysis of amide I bands indicated the percentages of area under peak (%) of main protein secondary structural components (*β*-turn, *α*-helix, and *β*-sheet) of rat's brain tissue in all groups administrated aluminium chloride in comparison to control.

Brain	*β*-Turn	*α*-Helix	*β*-Sheet
Control	25.3 ± 1	57.9 ± 2	16.8 ± 1
2-week AlCl_3_	23.4 ± 3	50.9 ± 1^†^	25.7 ± 1^†^
4-week AlCl_3_	29.5 ± 1^†^	43.9 ± 1^†^	26.6 ± 2^†^
6-week AlCl_3_	28.2 ± 1^†^	45.7 ± 2^†^	25.7 ± 1^†^
8-week AlCl_3_	37.7 ± 2^†^	37.4 ± 3^†^	24.9 ± 1^†^

^†^Statistically significant (*n* = 10, *p* < 0.05).

## Data Availability

Data is available upon request.
